# 

**Published:** 1951-01

**Authors:** 


### CONTENTS

| | Page |
|---|---|
| **Some Surgical Milestones.** By W. A. JACKMAN, *T.D.*, F.R.C.S. .. | 1 |
| **The XXXIX Long Fox Memorial Lecture.** By SIR WILLIAM SAVAGE, M.D., B.SC. .. .. .. .. .. .. .. | 9 |
| **Streptomycin.** By BERYL CORNER, M.D., M.R.C.P. AND SEYMOUR MASON, M.B., B.S., D.C.H. .. .. .. .. .. .. .. .. | 16 |
| **Keratosis Pharyngis.** By E. WATSON-WILLIAMS, *M.C.*, M.D., CH.M. .. | 23 |
| **Editorial:** THE PRESIDENCY; BLOOD TRANSFUSION .. .. .. | 25 |
| **In Memoriam: James Francis Blackett** .. .. .. .. | 26 |
| **Meetings of Societies** .. .. .. .. .. .. .. | 27 |
| **Local Medical Notes** .. .. .. .. .. .. .. | 32 |

